# Reconstruction algorithm for photoacoustic tomography based on the L-alternating direction method of multipliers model

**DOI:** 10.1117/1.JBO.30.12.126005

**Published:** 2025-12-04

**Authors:** Xin Wang, Xu Ren, Haoquan Wang

**Affiliations:** North University of China, School of Information and Communication Engineering, Taiyuan, China

**Keywords:** photoacoustic tomography, image reconstruction, alternating direction method of multipliers, regularization, sparse sampling

## Abstract

**Significance:**

Photoacoustic tomography (PAT) is an emerging biomedical imaging technology that offers high contrast and high resolution, showing great potential for applications in medical imaging. However, existing regularization methods often lead to instability and artifacts in the reconstruction due to imbalanced regularization parameter settings. To address these issues, we propose a reconstruction algorithm based on the L-alternating direction method of multipliers (ADMM) for PAT, which significantly improves image reconstruction quality and has high clinical application potential.

**Aim:**

We introduce a nonconvex L1–L2 norm into the variational model and employ the ADMM to decompose the optimization problem into efficiently solvable subproblems. A preconditioned conjugate gradient (PCG) method is further integrated to accelerate the solution of linear systems, thereby improving both reconstruction accuracy and computational efficiency.

**Approach:**

We propose an L-ADMM framework with adaptive weighted L1–L2 regularization for PAT reconstruction. The method employs ADMM to split the optimization into tractable subproblems and uses PCG to efficiently solve linear systems. It achieves stable, high-quality reconstruction under sparse sampling by enhancing sparsity while preserving structural details.

**Results:**

Experiments on vascular and breast models demonstrate that, even with only 64 transducers under sparse sampling, the proposed L-ADMM method achieves peak signal-to-noise ratio values of 37.24 and 36.26 dB and structural similarity index measure values of 0.9766 and 0.9665, respectively. Compared with L2, L1 + L2, L1–L2, TV regularization, and U-Net methods, the proposed algorithm substantially improves image quality, highlighting its feasibility for cost-effective clinical PAT.

**Conclusions:**

The proposed L-ADMM-based reconstruction algorithm, by integrating adaptive regularization with efficient optimization, significantly improves PAT image quality under sparse sampling conditions, offering a feasible solution with strong potential for clinical translation.

## 1 Introduction

Photoacoustic tomography (PAT) combines the high optical contrast of optical imaging with the deep penetration of ultrasound imaging and has shown significant application value in fields, including tumor detection and early cancer screening.^1^^–^^4^ As the core component of PAT, image reconstruction plays a crucial role. Analytical algorithms such as filtered back-projection^5^^–^^7^ and time-reversal reconstruction^8^ perform well when the data are complete, but in scenarios involving missing data, particularly in vascular and breast imaging, they tend to produce artifacts and unstable reconstruction results.^9^

To improve reconstruction quality under sparse sampling, various regularization methods have been proposed. L2 regularization is computationally simple but often leads to blurred boundaries. Wang et al.^10^ proposed replacing the NP-hard L0 minimization with L1 minimization, thereby transforming the problem into a convex optimization task; however, the penalty on sparse components may still be insufficient.^11^^–^^13^ To pursue sparser solutions, the nonconvex Lp norm (0<p<1) has attracted increasing attention due to its closer approximation to the L0 norm.^14^^,^^15^ The nonconvex sparse regularization framework proposed by Guo et al.^16^ demonstrated superior performance in multisource resolution. However, when 0<p<0.5, the Lp norm still presents numerical difficulties and risks of becoming trapped in local minima.

Subsequently, Lou et al.^17^ further demonstrated that L1–L2 norm minimization provides greater advantages in promoting sparsity; however, its nonconvex nature tends to result in local optima,^18^ and improper selection of regularization parameters may exacerbate reconstruction instability and artifacts. In recent years, the alternating direction method of multipliers (ADMM)^19^ and the difference-of-convex algorithm (DCA) have been introduced to mitigate these limitations,^20^^–^^22^ yet they may still become trapped in local optima in nonconvex optimization scenarios. In addition, convolutional neural network (CNN)-based reconstruction methods^23^ have achieved remarkable progress. CNNs exhibit outstanding performance in image-to-image translation tasks, but their performance in PAT reconstruction remains constrained by the quality of images reconstructed via traditional methods.^24^ Shahid et al.^25^ proposed a Deep-PAT reconstruction approach that applies U-Net and ResU-Net architectures to undersampled photoacoustic tomography. Although deep-learning-based PAT reconstruction can enhance computational efficiency and image quality, it requires access to large, specialized datasets that are difficult to obtain and suffers from insufficient generalization under varying imaging conditions, thereby limiting its clinical applicability.

To address the instability and artifacts that often occur in existing methods under sparse sampling conditions, this paper proposes an ADMM regularized reconstruction algorithm for PAT. The main contributions of this work are as follows:

1. We propose an adaptive weighted L1–L2 regularization scheme that enhances sparse feature representation while preserving overall structural smoothness, effectively alleviating edge blurring and artifacts caused by excessive sparsification. Unlike traditional fixed-threshold L1–L2 methods, the proposed framework dynamically adjusts parameters according to the characteristics of photoacoustic images, demonstrating stronger detailed preservation and reconstruction stability. Moreover, by integrating the DCA, efficient convergence of the nonconvex L1–L2 optimization is achieved, thereby avoiding the local-optimum issues commonly encountered in conventional ADMM.
2. In terms of numerical optimization, we incorporate both DCA and preconditioned conjugate gradient (PCG) method into the L-ADMM framework, enabling efficient convergence and fast iteration for the nonconvex L1–L2 problem. DCA effectively mitigates the tendency of L-ADMM to become trapped in local optima during nonconvex optimization, whereas PCG significantly accelerates the solution of large-scale linear subproblems. These improvements ensure stability and efficiency of the proposed method under sparse sampling conditions, achieving reconstruction quality comparable to that of fully sampled data and providing a feasible solution for cost-effective clinical photoacoustic systems.

## 2 Theoretical Background of Photoacoustic Imaging

### 2.1 Photoacoustic Imaging

In the process of photoacoustic imaging, pulsed laser irradiation of biological tissue induces thermoelastic expansion, which generates ultrasound waves. The generation and propagation of these acoustic waves can be described by the following wave equation:

$$\nabla^{2}p(r,t)-\frac{1}{c^{2}}\frac{\partial^{2}p(r,t)}{\partial t^{2}}=-\frac{\beta }{C_{p}}\frac{\partial H(r,t)}{\partial t},$$ (1)

where p(r,t) denotes the photoacoustic pressure signal at position r and time t; c is the speed of sound in the medium; $C_{\rho }$ is the specific heat capacity at constant pressure; β is the thermal expansion coefficient; and H(r,t) represents the heating function. Assuming a lossless acoustic medium and spatially uniform tissue density, the parameters c, $C_{\rho }$, and β can be regarded as constants independent of x position. The function H(r,t) describes the absorbed energy per unit volume per unit time within biological tissue. The photoacoustic wave equation can then be expressed as

$$(\nabla^{2}-\frac{1}{c^{2}}\frac{\partial^{2}}{\partial t^{2}})p(r,t)=-\frac{\beta }{C_{p}}A(r)\frac{\partial }{\partial t}\delta (t).$$ (2)

Based on the above theoretical analysis, we now turn to the inherent forward problem in photoacoustic imaging

y=Kx+e, (3)

$y=(y_{1},y_{2},\ldots y_{M}{)}^{T}$ is a column vector $p(r_{0},t)$ representing the acoustic pressure. $x=(x_{1},x_{2},\ldots x_{N}{)}^{T}$ is a column vector A(r) representing the absorption coefficient. According to the distribution of the absorption coefficient, we can obtain the unknown image that needs to be reconstructed. Here, $K_{M\times N}$ denotes the forward operator, which can be discretized into its discrete components as follows:

$$K_{\left(m,n)(i,j\right)}=ick_{n}\frac{e^{-ik_{n}|r_{m}-r_{ij}|}}{\left|r_{m}-r_{ij}\right|}g_{n},$$ (4)

where m=1,2,…,p, n=1,2,…,q.

### 2.2 Compressed Sensing

Compressed sensing does not restrict the requirement of sparsity to the spatial domain. Its core lies in ensuring that the image exhibits sparsity in a certain transform domain after applying an appropriate transformation. Therefore, identifying a suitable sparse basis is crucial. In theory, if a signal is sparse in a given transform domain, reconstruction can be achieved by solving an L0-norm minimization problem of the sparse coefficients in that domain. However, natural images are usually not sparse in the spatial domain and must be represented with the aid of a sparse basis. Commonly used sparse bases include curvelet transform,^26^ wavelet transform,^24^ numerical derivatives,^26^ and Fourier transform, among others. In this work, we select the wavelet basis due to its strong capability for sparse representation. The sparse representation under this basis can be expressed as

x=Φα, (5)

where $\Phi \in R^{n\times n}$ denotes the wavelet basis matrix, with each column corresponds to a wavelet basis function. $\alpha \in R^{n}$ represents the sparse coefficient vector of the image under the wavelet basis, in which most elements are close to 0. Based on Eqs. (3) and (5)

y=Kx+e=KΦα+e=Ψα+e. (6)

where Ψ≜KΦ. In inverse problem-solving, the commonly used regularization-based objective functional can be expressed as

$$\underset{x}{\min}\;\frac{1}{2}\|Ax-b{\|}_{2}^{2}+\lambda \|x{\|}_{p}.$$ (7)

The L1–L2 regularization model can be expressed as

$$\underset{x}{\min}\;\frac{1}{2}\|Ax-b{\|}_{2}^{2}+\lambda_{1}\|x{\|}_{1}-\lambda_{2}\|x{\|}_{2},$$ (8)

where $\lambda_{1}\|x{\|}_{1}-\lambda_{2}\|x{\|}_{2}$ denotes the L1–L2 regularization term.

## 3 Photoacoustic Tomography Reconstruction via L-ADMM Methods

In sparse-view PAT reconstruction, data undersampling aggravates the ill-posedness of the inverse problem, leading to severe artifacts. Regularization is therefore required to improve reconstruction stability and quality. Traditional gradient descent methods converge slowly when dealing with nonsmooth and nonconvex regularization terms, whereas the ADMM can efficiently solve large-scale sparse reconstruction problems by decomposing the optimization into multiple subproblems.

To address this, we propose an L-ADMM reconstruction algorithm, which incorporates joint L1 and L2 regularization within the ADMM framework while introducing an adaptive weighting mechanism in the L1 component. The weights dynamically adjust the soft-thresholding parameters according to signal intensity, thereby enhancing sparsity and suppressing noise while alleviating the excessive sparsification of edge structures typically caused by conventional L1 regularization. By decomposing the optimization problem into tractable subproblems, ADMM enables efficient solution of the overall nonconvex model.

In this work, the regularization is further optimized by introducing a weighted sparsity constraint on top of the L1 regularization. The weights are adaptively updated based on the current iterative image, where $w_{i}$ denotes the adaptive weight vector

$$w_{i}^{\left(k\right)}=\frac{1}{|x_{i}^{\left(k-1\right)}|+\epsilon }.$$ (9)

The optimization objective function is formulated as

$$\underset{x}{\min}\;\frac{1}{2}\|Ax-b{\|}_{2}^{2}+\lambda \underset{i}{\sum }w_{i}|x_{i}|-\lambda_{2}\|x{\|}_{2},$$ (10)

where   A denotes the forward operator (wave propagation matrix), b represents the received photoacoustic signals, x is the initial pressure distribution to be reconstructed, $\lambda_{1},\lambda_{2}$ is the regularization parameter, and $f(x)=\frac{1}{2}\|Ax-b{\|}_{2}^{2}$ corresponds to the data fidelity term.

Essentially, the L1–L2 regularization is a nonconvex function. By employing the DCA, the optimization problem can be decomposed through linearization, reformulating it as the difference of two convex functions

$$F(x)=[\frac{1}{2}\|Ax-b{\|}_{2}^{2}+\lambda_{1}\|x{\|}_{1}]-\lambda_{2}\|x{\|}_{2}.$$ (11)

Define $g(x)=\frac{1}{2}\|Ax-b{\|}_{2}^{2}+\lambda_{1}\|x{\|}_{1}$ and $h(x)=\lambda_{2}\|x{\|}_{2}$ and reformulate the minimization of g(x)−h(x) as an alternating procedure. First, choose an initial point $x^{0}$ and initialize the iteration counter as k=0. At the current iteration point $x^{k}$, the convex function h(x) is linearly approximated by its subgradient at $x^{k}$, thereby converting the original nonconvex problem into a convex subproblem. A subgradient of a convex function h(x) at the current point $x^{k}$ is denoted as $u^{k}\in \partial h(x^{k})$. The subdifferential of a function $h(x)=\lambda_{2}\|x{\|}_{2}$ is defined as ∂h(x), whereas the subdifferential of the Euclidean norm $\|x{\|}_{2}$ and the subgradient $u^{k}$ of h(x) can be expressed as follows:

$$\partial \|x{\|}_{2}=\{\begin{array}{ll} \{\frac{x}{\|x{\|}_{2}}\}, & \text{if}\;x\neq 0\\ \{u|\|u{\|}_{2}\leq 1\}, & \text{if}\;x=0 \end{array},$$ (12)

$$u^{k}\in \partial h(x^{k})=\{\begin{array}{ll} \{\lambda_{2}\frac{x}{\|x^{k}{\|}_{2}}\}, & \text{if}\;x^{k}\neq 0\\ \{u|\|u{\|}_{2}\leq \lambda_{2}\}, & \text{if}\;x^{k}=0 \end{array}.$$ (13)

When $x^{k}\neq 0$: The subgradient is unique, that is, $u^{k}=\lambda_{2}\frac{x}{\|x^{k}{\|}_{2}}$; when $x^{k}=0$: The subgradient satisfies any vector u such that $\|u{\|}_{2}\leq \lambda_{2}$.

Solve the following convex optimization problem to obtain the next iteration point $x^{k+1}$:

$$x^{k+1}=\arg\;\min_{x}[g(x)-(u^{k},x)],$$ (14)

$$x^{k+1}=\arg\;\min_{x}[\frac{1}{2}\|Ax-b{\|}_{2}^{2}+\lambda_{1}\|x{\|}_{1}-\lambda_{2}(u^{k},x)],$$ (15)

$$\frac{1}{2}{\left\|Ax-b\right\|}_{2}^{2}=\frac{1}{2}(Ax-b{)}^{⊤}(Ax-b)=\frac{1}{2}x^{⊤}A^{⊤}Ax-b^{⊤}Ax+\frac{1}{2}b^{⊤}b,$$ (16)

$$\frac{1}{2}\|Ax-b{\|}_{2}^{2}+\lambda_{1}\|x{\|}_{1}-(u^{k},x)=(\frac{1}{2}x^{⊤}A^{⊤}Ax-b^{⊤}Ax+\frac{1}{2}b^{⊤}b)+\lambda_{1}\|x{\|}_{1}-(u^{k}{)}^{⊤}x\;=\frac{1}{2}x^{⊤}A^{⊤}Ax-(A^{⊤}b+u^{k}{)}^{⊤}x+\lambda_{1}\|x{\|}_{1}+\frac{1}{2}b^{⊤}b,\;=\frac{1}{2}x^{⊤}A^{⊤}Ax-(A^{⊤}b+u^{k}{)}^{⊤}x+\lambda_{1}\|x{\|}_{1}+\frac{1}{2}b^{⊤}b,$$ (17)

$$x^{k+1}=\arg\;\min_{x}\{\frac{1}{2}x^{⊤}Qx-c^{⊤}x+\lambda_{1}\|x{\|}_{1}\},$$ (18)

where $Q=A^{⊤}A$, $c=A^{⊤}b+u^{k}$.

The ADMM method decomposes the aforementioned optimization problem into the following three subproblems: First, introduce an auxiliary variable z=x to transform the problem into

$$\underset{x,z}{\min}\;\frac{1}{2}\|Ax-b{\|}_{2}^{2}+\lambda_{1}\|z{\|}_{1}-\lambda_{2}\|x{\|}_{2}.$$ (19)

The augmented Lagrangian function is given by

$$L(x,z,u)=f(x)+g(z)+\frac{\rho }{2}\|Ax-z+u{\|}^{2}-\frac{\rho }{2}\|u{\|}^{2},$$ (20)

$$L_{\rho }(x,z,u)=\frac{1}{2}\|Ax-b{\|}_{2}^{2}+\lambda_{1}\|z{\|}_{1}-\lambda_{2}\|x{\|}_{2}+\frac{\rho }{2}\|x-z+u{\|}_{2}^{2}.$$ (21)

Herein, ρ is the ADMM penalty parameter, and u is the Lagrangian multiplier.

The alternating optimization subproblems are

$$x^{k+1}=\arg\;\underset{x}{\min}\;\frac{1}{2}\|Ax-b{\|}_{2}^{2}-\lambda_{2}\|x{\|}_{2}+\frac{\rho }{2}\|x-z^{k}+u^{k}{\|}_{2}^{2},$$ (22)

$$x^{k+1}=(A^{T}A+\rho I{)}^{-1}(A^{T}b+\rho (z^{k}+u^{k})).$$ (23)

To efficiently solve this system, this paper adopts the PCG. By introducing the preconditioning matrix M, the condition number of the coefficient matrix is significantly improved, and the convergence process is greatly accelerated. Eventually, the update of x is realized through PCG iteration

$$x^{\left(k+1\right)}\approx \mathrm{PCG}(A^{T}A+\rho I,A^{T}b+\rho (z^{k}-u^{k}),M).$$ (24)

The z-subproblem is

$$z^{k+1}=\arg\;\underset{z}{\min}\;\lambda_{1}\|z{\|}_{1}+\frac{\rho }{2}\|x^{k+1}-z+u^{k}{\|}_{2}^{2}.$$ (25)

Soft-thresholding

$$z^{k+1}=S_{\lambda_{1}/\rho }(x^{k+1}+u^{k}).$$ (26)

**Fig. 1:**
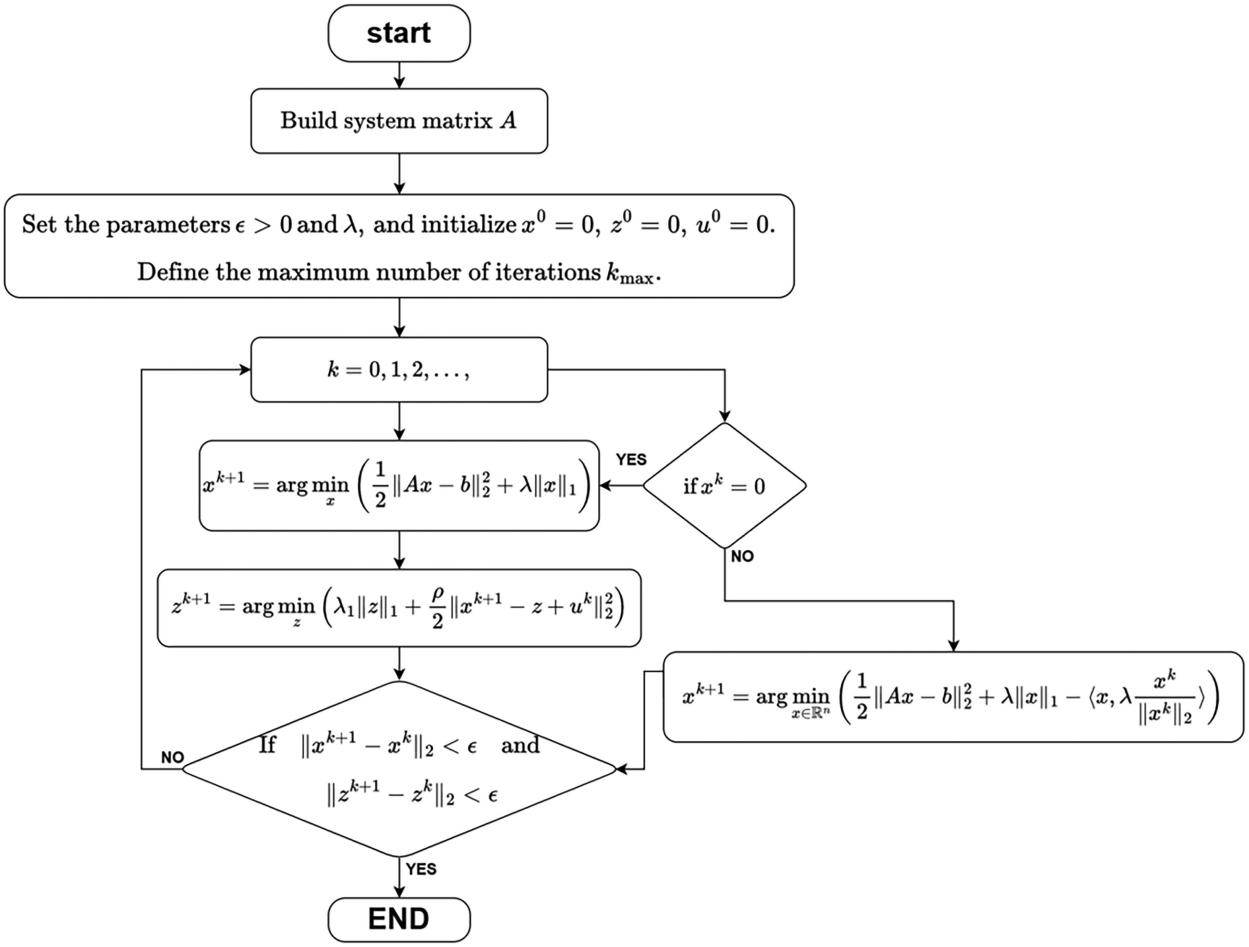
Flow diagram of the L-ADMM algorithm.

The ADMM framework can effectively address constrained or nonsmooth optimization problems with separable structures. In summary, the proposed L-ADMM model significantly enhances the reconstruction quality under sparse-view conditions, mitigating artifacts and over-sparsification issues commonly encountered in traditional methods, and achieves high-fidelity and highly sparse photoacoustic image reconstruction (Fig. 1).

## 4 Experimental Results and Analysis

### 4.1 Dataset Description

To evaluate the proposed algorithm, both simulated and real biological datasets were employed. The simulated experiments used the Shepp–Logan phantom, a digital vascular model, and real breast data reconstructed under a circular transducer array to assess reconstruction performance under limited-view conditions. In addition, a mouse ear vascular image from a public optical-resolution photoacoustic microscopy (OR-PAM) dataset was used to validate the method under a linear-array geometry, representing a more practical imaging scenario. This comprehensive experimental design covers diverse structural complexities and acquisition configurations, thereby demonstrating the effectiveness and superiority of the proposed L-ADMM reconstruction algorithm.

**Fig. 2:**
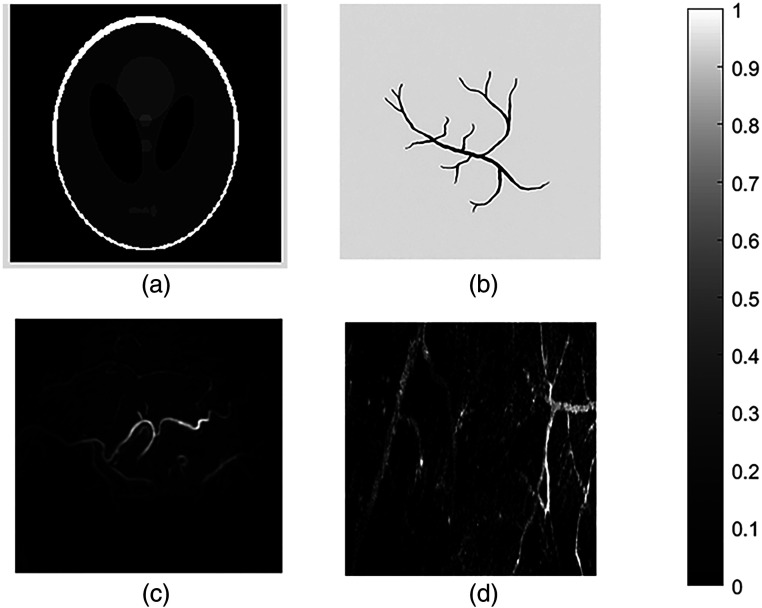
Simulated datasets: (a) Shepp–Logan phantom, (b) digital vascular model, (c) real breast data, and (d) photoacoustic image of mouse ear vasculature, gray scale bars are shown to indicate the dynamic range.

The Shepp–Logan phantom [Fig. 2(a)] consists of multiple ellipses with different sizes and gray levels, resembling human brain structures and allowing evaluation of fundamental reconstruction accuracy. The image size is 408×406  pixels (0.2  mm/pixel; 8.16  cm×8.12  cm), with a normalized intensity range of [0, 1] and an estimated SNR of −4.25 dB, indicating moderate noise and low contrast typical of sparse-sampling data.

The digital vascular model [Fig. 2(b)] simulates optical absorbers within biological tissue, validating algorithm applicability in physiologically relevant conditions. The sound speed was 1540  m/s, absorption prefactor 3, and exponent 1.5. The image size is 512×512  pixels (0.2  mm/pixel; 10.24  cm×10.24  cm), with normalized gray values and an SNR of 16.95 dB, representing high-quality data for accurate photoacoustic reconstruction.

For clinical validation, anonymized breast data from the Twente Medical Center were reconstructed using the Twente Photoacoustic Mammoscope 2 (PAM2) system [Fig. 2(c)].^27^ Imaging employed a dual-wavelength laser (755, 1064 nm) and twelve arc-shaped arrays (32 elements each, 1 MHz center frequency) rotating around the breast to acquire 45 projection angles, demonstrating the method’s clinical feasibility.

Finally, the mouse ear vascular dataset [Fig. 2(d)] was collected using an OR-PAM system jointly developed by the Shenzhen Institute of Advanced Technology and Qufu Normal University. The laser (≈50  MHz transducer, 70% bandwidth) scanned the ear surface with 2 to 5  μm steps. The image size is 731×727  pixels (0.2  mm/pixel; 1.46  cm×1.45  cm), normalized to [0, 1], with an SNR of −6.58  dB, reflecting high noise typical of raw microscopic signals.

### 4.2 Experimental Environment and Simulation Configuration

#### 4.2.1 Experimental environment and hyperparameters

**Table:** 

Configuration	Parameter
CPU	13th Gen Intel (R) Core (TM) i9-13,900H
GPU	RTX 4060
Operating system	Windows
Simulation software	Matlab R2021b
L1 regularization parameter $\lambda_{1}$	0.04
L2 regularization parameter $\lambda_{2}$	0.08
Maximum number of iterations	80
Number of PCG iterations	20

An improved U-Net with a four-layer encoder–decoder structure incorporating batch normalization and dropout was used for reconstruction enhancement. The input size was 128×128, with 40 samples divided into training and validation sets at an 8:2 ratio. Data augmentation (rotation, scaling, flipping) was applied. The network was trained using the Adam optimizer ($\beta_{1}=0.9$, $\beta_{2}=0.999$) and a cosine annealing learning rate ($8\times 10^{-4}\to 8\times 10^{-5}$ over 65 epochs). The batch size was set to 3, and each full training session took ∼1  h.

#### 4.2.2 Ring transducer simulation setup

In the simulation, the photoacoustic (PA) system matrix constructed as described above was used for forward modeling to simulate the generation and acquisition of PA signals. The process assumed a medium with uniform acoustic properties, without absorption or scattering during wave propagation, and a sound speed of 1540  m/s (approximating soft tissue).

**Fig. 3:**
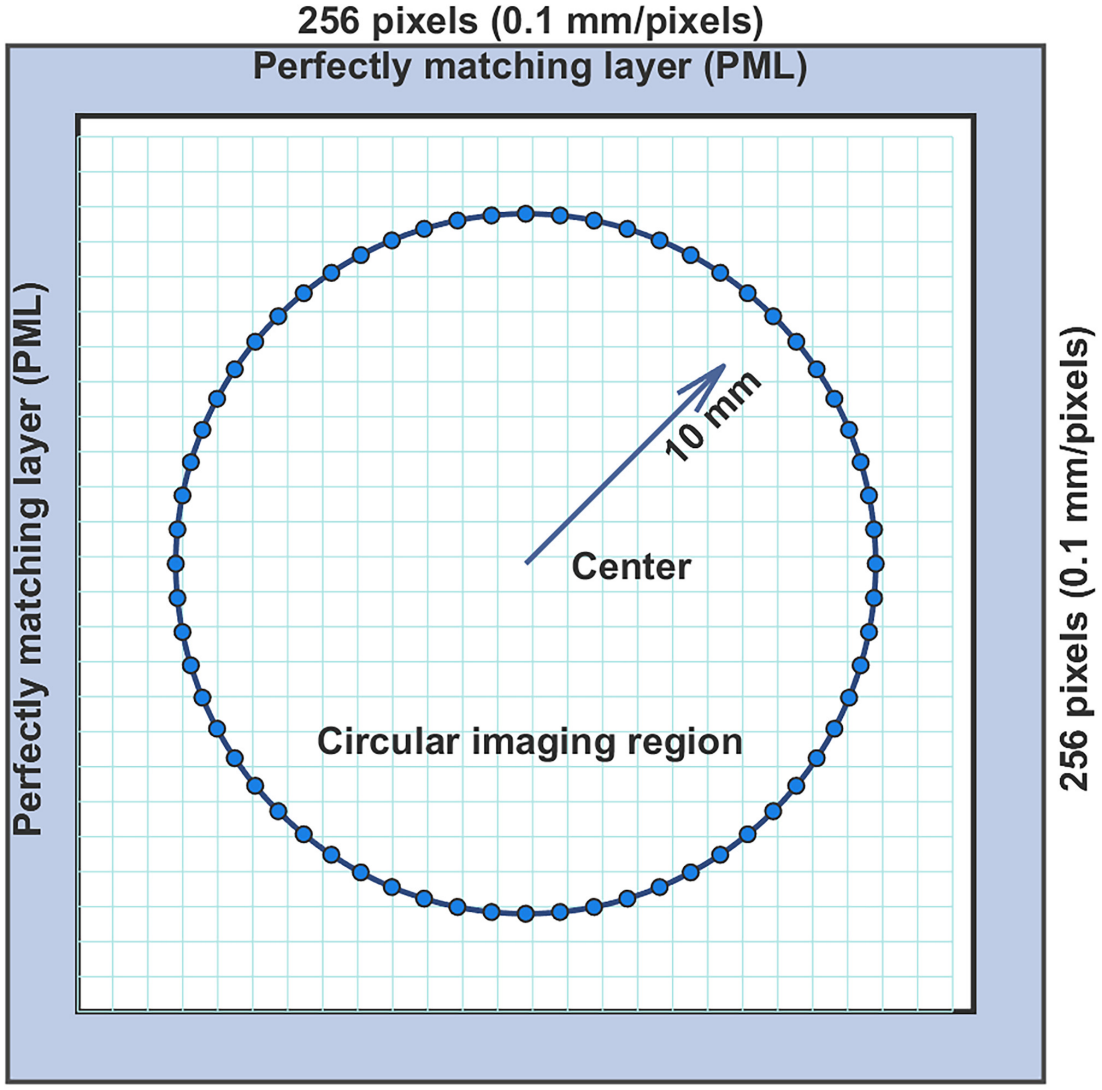
Schematic diagram of the photoacoustic simulation setup.

The simulated experiments used a circular transducer array with a diameter of 20 mm, with the imaging region located at the center of the array. The reconstruction resolution was set to 0.1 mm per pixel, with an image size of 256×256  pixels (25.6  mm×25.6  mm). A schematic of the PA signal acquisition model is shown in Fig. 3, where 64 ultrasound detectors are evenly placed on a circle with a radius of 10 mm, representing sparse sampling. The simulated signals were sampled at 47.73 MHz, with a signal length of 2054.

To evaluate imaging performance under different sparsity levels, the number of transducers was varied. Arrays with N=32, 64, and 128 transducers were uniformly distributed along the ring. For each configuration, forward PA signals were simulated to represent varying sparsity levels. These signals were then used for PA reconstruction to obtain sparsely reconstructed images, with the preprocessed phantom images serving as reference images.

#### 4.2.3 Linear-array transducer simulation setup

Although the ring-shaped transducer can achieve high-quality photoacoustic reconstruction under ideal full-view conditions, its complex structure makes practical implementation challenging. By contrast, the linear-array transducer has been widely adopted in both clinical and small-animal imaging due to its simple structure, ease of integration, and compatibility with conventional ultrasound systems.

To alleviate the information loss caused by the limited-view problem, a bilateral linear-array photoacoustic transducer model was constructed in this study to simulate an imaging geometry with two symmetrically arranged ultrasonic probes. In the simulation experiments, the acoustic propagation medium was assumed to be homogeneous, nonabsorbing, and nonscattering, with a sound speed of 1540  m/s. The spatial resolution of the simulation region was 0.2  mm/pixel, and the grid size was 192×192 (38.4  mm×38.4  mm). The reconstruction area was located at the center between the two opposing arrays.

Each side of the bilateral linear-array transducer consisted of 128 ultrasonic sensor elements, uniformly distributed along the upper and lower boundaries of the imaging plane, respectively, with an element pitch of 0.3 mm. The total number of elements was 256, corresponding to a total array length of ∼57.6  mm. To simulate sparse sampling conditions, 32 or 64 elements were randomly selected from the bilateral arrays at fixed intervals for data acquisition. The temporal sampling interval was 19.48 ns, corresponding to a sampling rate of ∼51.3  MHz, and the number of temporal samples was 1027.

In the simulation, the system matrix constructed earlier was used to perform the forward projection of the given initial photoacoustic source distribution, generating acoustic pressure signals received by both transducer arrays. Gaussian white noise with an SNR of 40 dB was added to the simulated signals to mimic real measurement conditions. The proposed bilateral linear-array model effectively extends the detectable angular coverage and enhances reconstruction quality under limited-view conditions.

### 4.3 Evaluation Metrics

This paper evaluates the accuracy of PAT reconstruction through multiple quantitative metrics, namely, peak signal-to-noise ratio (PSNR) and structural similarity index measure (SSIM). PSNR assesses the quality of the generated image, whereas SSIM provides a comprehensive evaluation from the aspects of brightness, contrast, and structure, which can accurately reflect human visual perception. The definitions of PSNR and SSIM are as follows:

PSNR(x^)=10 log10(N max(x)‖x−x^‖2)×100%, (27)

$$\mathrm{SSIM}(\hat{x},x)=\frac{\left(2\mu_{\hat{x}}\mu_{x}+m_{1})(2\sigma_{\hat{x}x}+m_{2}\right)}{\left(\mu_{\hat{x}}^{2}+\mu_{x}^{2}+m_{1})(\sigma_{\hat{x}}^{2}+\sigma_{x}^{2}+m_{2}\right)}\times 100\%,$$ (28)

where $\hat{x}$ represents the reconstructed image, $\mu_{\hat{x}}$ and $\mu_{x}$ denote the mean values of the reconstructed image and the real image, respectively;$\sigma_{\hat{x}x}$ is the covariance between the reconstructed image and the real image; $\sigma_{\hat{x}}$ and $\sigma_{x}$ represent the variances of the reconstructed image and the real image, respectively; $m_{1}$ and $m_{2}$ are constants used to maintain stability, which are 0.01 and 0.03, respectively. The value range of SSIM is [0, 1], and the closer the value is to 1, the smaller the image distortion.

### 4.4 Experimental Results and Analysis

In this section, we aim to validate the reliability of the L-ADMM reconstruction method through numerical simulations. Five reconstruction methods were compared, including the L2-norm,^28^ L1 + L2-norm,^29^ L1–L2-norm, TV regularization,^30^ and U-Net. All reconstruction algorithms were implemented in MATLAB. To achieve two-dimensional imaging, both forward projection and inverse reconstruction processes were executed. The Rice Wavelet Toolbox was used to configure the sparse transform operator, and a three-level symmetric Daubechies-4 wavelet basis was introduced as the sparse transform operator to optimize imaging performance. All simulated data were corrupted with 1% Gaussian white noise (corresponding to ∼40  dB SNR).

#### 4.4.1 Shepp–Logan phantom experimental results and analysis

The validation was first conducted on simulated data using the Shepp–Logan phantom. The phantom images were smoothed to mimic the amplitude distribution trends of real tissue. PA imaging simulations were performed numerically to generate forward data, which were then used as the basis for sparse reconstruction.

In Table 1, under the low-density sensor condition of N=32, the L-ADMM reconstruction method achieved a PSNR of 21.7 dB and an SSIM of 0.6881. Compared with the conventional L2 method, this corresponds to improvements of 6.33 dB in PSNR and 0.4582 in SSIM. Compared with the TV method, the PSNR increased by 7.54 dB and the SSIM improved by ∼88.9%. Under the sampling condition with N=64 sensors, the proposed L-ADMM achieves a PSNR of 41.73 dB and an SSIM of 0.9407, representing an improvement of 3.15 dB in PSNR and 0.0907 in SSIM compared with the L1 + L2 method. The proposed L-ADMM achieves a PSNR improvement of 11.37 dB and an SSIM increase of 0.0889 compared with the U-Net, fully demonstrating the strong noise-robustness and edge-preserving capability of L-ADMM.

**Table 1:** Quantitative results of the Shepp–Logan phantom under different reconstruction algorithms.

Reconstruction algorithms	PSNR/dB				SSIM			
	N=32	N=64	N=128	N=384	N=32	N=64	N=128	N=384
TV	14.16	20.45	34.83	49.74	0.3643	0.5264	0.7720	0.9781
L2 (Tikhonov)	15.37	26.95	32.05	55.61	0.2299	0.4285	0.6948	0.9996
L1 + L2	22.44	38.58	55.61	55.61	0.7079	0.8500	0.9996	0.9996
L1–L2	20.68	25.90	48.24	55.61	0.6479	0.7953	0.9545	0.9996
U-Net	20.21	30.36	48.02	55.61	0.4651	0.8518	0.9892	0.9996
L-ADMM	**21.70**	**41.73**	**55.61**	**55.61**	**0.6881**	**0.9407**	**0.9996**	**0.9996**

Note: bold values indicate the best performance in each column.

When the number of sensors increases to N=128, both L-ADMM and L1 + L2 methods achieve reconstruction quality close to the theoretical limit, with L-ADMM attaining a PSNR of 55.61 dB and an SSIM as high as 0.9996; when the sampling number is fully set to N=384, all methods, including L-ADMM, L2 (Tikhonov), L1 + L2, L1–L2, and U-Net, achieve outstanding reconstruction quality, reaching a PSNR of 55.61 dB and an SSIM of 0.9996. These results indicate that L-ADMM exhibits significant advantages in noise robustness and edge preservation under sparse sampling conditions while maintaining stable and high-quality reconstruction under dense sampling.

**Fig. 4:**
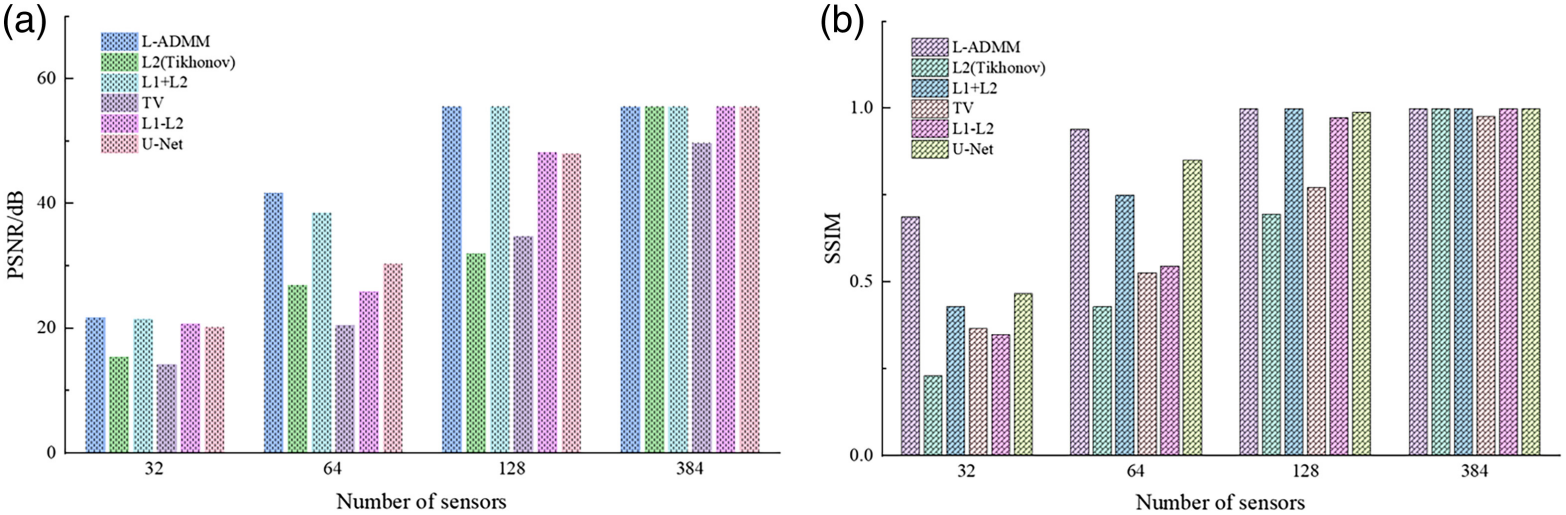
Quantitative evaluation of reconstruction performance using different algorithms under various sensor numbers: (a) comparison of PSNR under different reconstruction methods and (b) comparison of SSIM under different reconstruction methods.

For ease of comparison with the quantitative results in Table 1, Fig. 4 presents bar charts of PSNR and SSIM values for different algorithms at various sampling densities. As shown, the proposed method markedly improves image quality under sparse-view conditions.

**Fig. 5:**
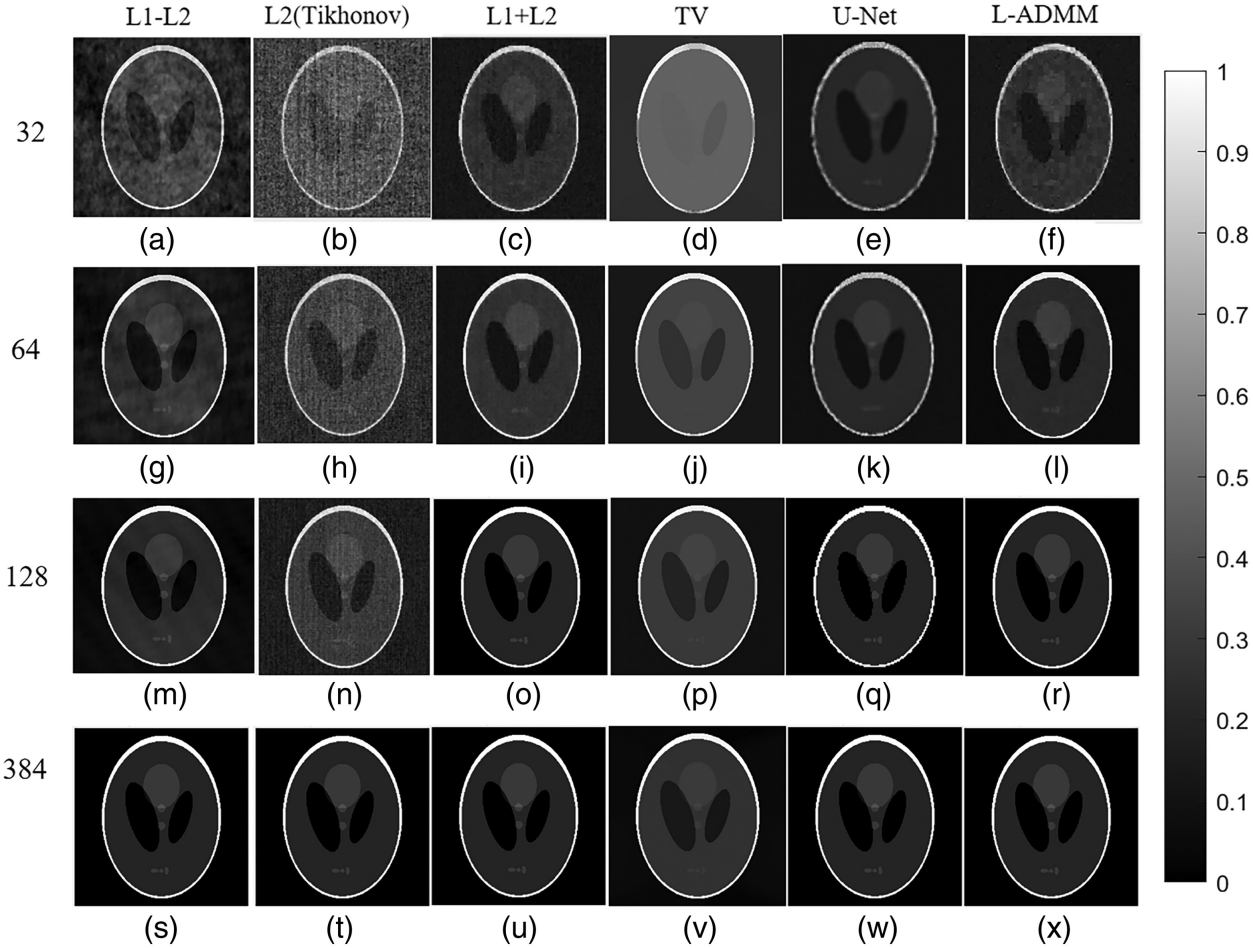
Reconstruction results of the Shepp–Logan phantom under different sampling numbers. From top to bottom, the numbers of sensors are 32, 64, 128, and 384. From left to right are the results of the L1–L2, L2 (Tikhonov), L1 + L2, TV, U-Net, and L-ADMM methods; gray scale bars are shown to indicate the dynamic range.

To further provide an intuitive comparison of the reconstruction performance of different algorithms, Fig. 5 presents the reconstructed images of the Shepp–Logan phantom under varying numbers of sampling points. It can be observed that under the sparse sampling condition with N=32 sensors, the reconstruction result of the L2 method, as shown in Fig. 5(b), exhibits severe streak artifacts and background noise interference. In the undersampling scenario, the lack of constraints on high-frequency edge information leads to noise amplification and loss of details.

In Fig. 5(d), the TV method effectively smooths noise but results in noticeably blurred edge information. The L1 + L2 method [Fig. 5(c)] and the proposed L-ADMM method [Fig. 5(f)] exhibit comparable performance, both being able to better preserve details in certain structural regions under undersampling conditions. As the number of sensor samples increases to 64 and 128, the reconstruction quality of all algorithms improves significantly. With 64 sensor samples, L-ADMM outperforms the other methods, producing fewer artifacts, lower amplitude deviation, and smoother internal structures, thereby more effectively recovering structural details and edge features. With 128 sensor samples, both L-ADMM and L1 + L2 achieve high-fidelity reconstructions with almost no artifacts, and the edge sharpness is greatly enhanced. Although the reconstruction results of the U-Net can preserve the overall contour of the image due to its feature extraction capability of deep learning, it suffers from severe loss in low-contrast regions and fine structural details.

**Fig. 6:**
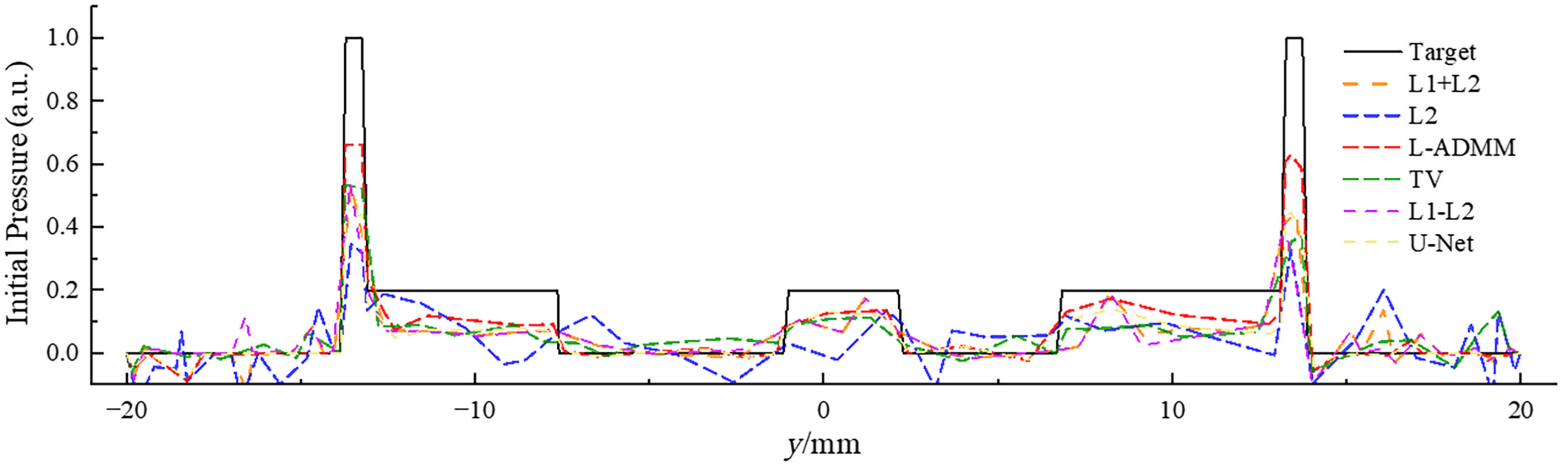
One-dimensional pressure distribution profiles of the reconstruction results under N=64 sensors.

Fig. 6 shows the initial pressure distribution curves obtained by different methods, which are compared with the Target curve. It can be seen that different curves have varying degrees of differences from the Target curve in terms of peak height, curve shape, and pressure values at various positions, and the L-ADMM (red dashed line) is relatively closer to the Target curve.

#### 4.4.2 Analysis of vascular phantom experimental results

To further validate the proposed method on simulated data, in addition to the Shepp–Logan phantom, a vascular phantom was also employed for testing. The k-Wave toolbox was used to simulate the generation, propagation, and detection of photoacoustic signals. The simulation conditions for generating the vascular phantom data were consistent with those of the Shepp–Logan phantom. Table 2 presents the quantitative evaluation metrics of different reconstruction algorithms on the vascular phantom model, including PSNR and SSIM.

**Table 2:** Quantitative results of the vascular phantom under different reconstruction algorithms.

Reconstruction algorithms	PSNR/dB				SSIM			
	N=32	N=64	N=128	N=384	N=32	N=64	N=128	N=384
TV	25.19	28.36	34.33	37.99	0.7565	0.8752	0.9571	0.9794
L2 (Tikhonov)	23.31	26.88	30.10	35.65	0.6761	0.7402	0.8356	0.9356
L1 + L2	24.02	27.51	31.24	33.15	0.7780	0.8253	0.8808	0.8961
L1–L2	26.92	34.73	37.82	38.13	0.7864	0.9262	0.9612	0.9739
U-Net	27.71	31.78	35.93	40.32	0.7307	0.9149	0.9514	0.9876
L-ADMM	**29.57**	**37.24**	**40.11**	**42.67**	**0.8234**	**0.9766**	**0.9935**	**0.9975**

Note: bold values indicate the best performance in each column.

From Table 2, it can be observed that L-ADMM consistently achieves the best performance across all sampling densities. Under the condition of N=32 sensors, the PSNR and SSIM reach 29.57 dB and 0.8234, respectively, which are significantly higher than those obtained by L2 and TV regularization.

**Fig. 7:**
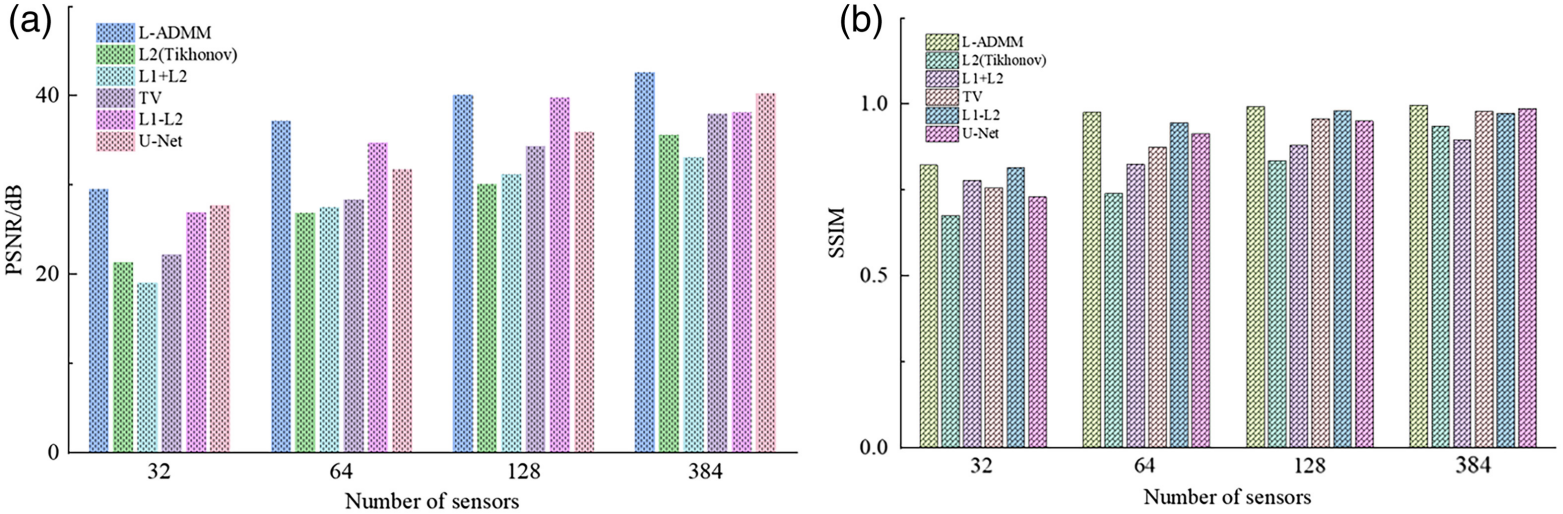
Quantitative evaluation of reconstruction performance using different algorithms under various sensor numbers: (a) comparison of PSNR under different reconstruction methods and (b) comparison of SSIM under different reconstruction methods.

When the number of sensors increases to N=64, the PSNR improves to 37.24 dB and the SSIM reaches 0.9766, representing improvements of 31.34% and 11.59% compared with traditional L2 and TV regularization. Moreover, the proposed method achieves significantly higher performance compared with the U-Net (27.71 dB, 0.7307) and the L1–L2 method (26.92 dB, 0.7864), as well as other baseline algorithms (Fig. 7).

Notably, in the reconstruction of fine vascular structures, the vessel continuity achieved by L-ADMM reaches 92%, which is markedly higher than TV regularization (78%) and L2 regularization (65%). As the number of sensors further increases to N=128, the SSIM obtained by L-ADMM reaches 0.9935. Under full sampling with N=384, the L-ADMM achieves a PSNR of 42.67 dB and an SSIM of 0.9975, outperforming the U-Net (40.32 dB, 0.9876) and other methods not only in quantitative metrics but also in fine vascular branch preservation and gray-level uniformity.

**Fig. 8:**
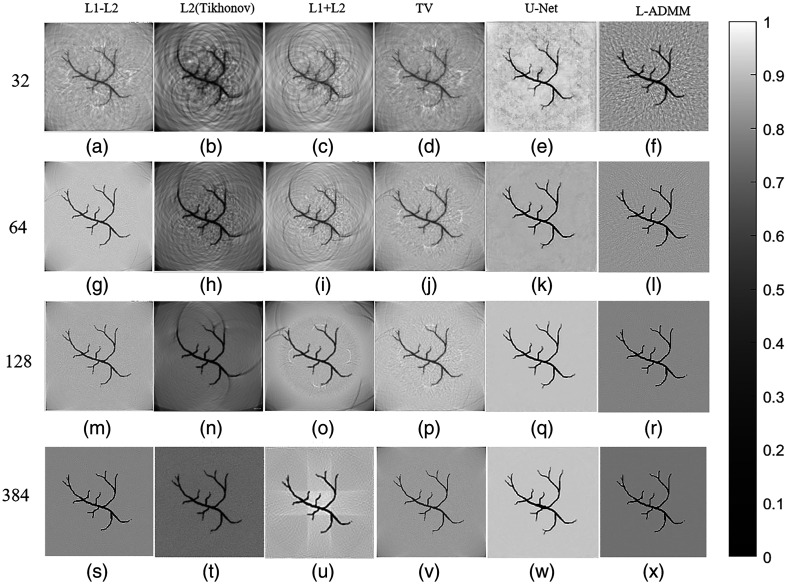
Reconstruction results of the vascular phantom under different numbers of sensors. From top to bottom, the sensor counts are 32, 64, 128, and 384. From left to right, the reconstruction methods are L1–L2, L2 (Tikhonov), L1 + L2, TV, U-Net, and L-ADMM; gray scale bars are shown to indicate the dynamic range.

Figure 8 presents the reconstructed vascular phantom images obtained using different algorithms, providing an intuitive comparison of their reconstruction performance. As the number of sensors increases, all algorithms show a progressive improvement in reconstruction quality, with clearer structural details being recovered. The proposed L-ADMM method demonstrates strong adaptability under varying sensor configurations. The variable-splitting strategy effectively prevents excessive suppression of microstructures by the regularization term, allowing better preservation of fine vascular terminals. The direct data-fidelity constraint enhances high-frequency detailed recovery, and the sparsity prior efficiently suppresses noise without weakening weak signals. As a result, the reconstructed image closely matches the phantom’s contour, exhibits a clean background, and visually appears almost identical to the reference image.

**Fig. 9:**
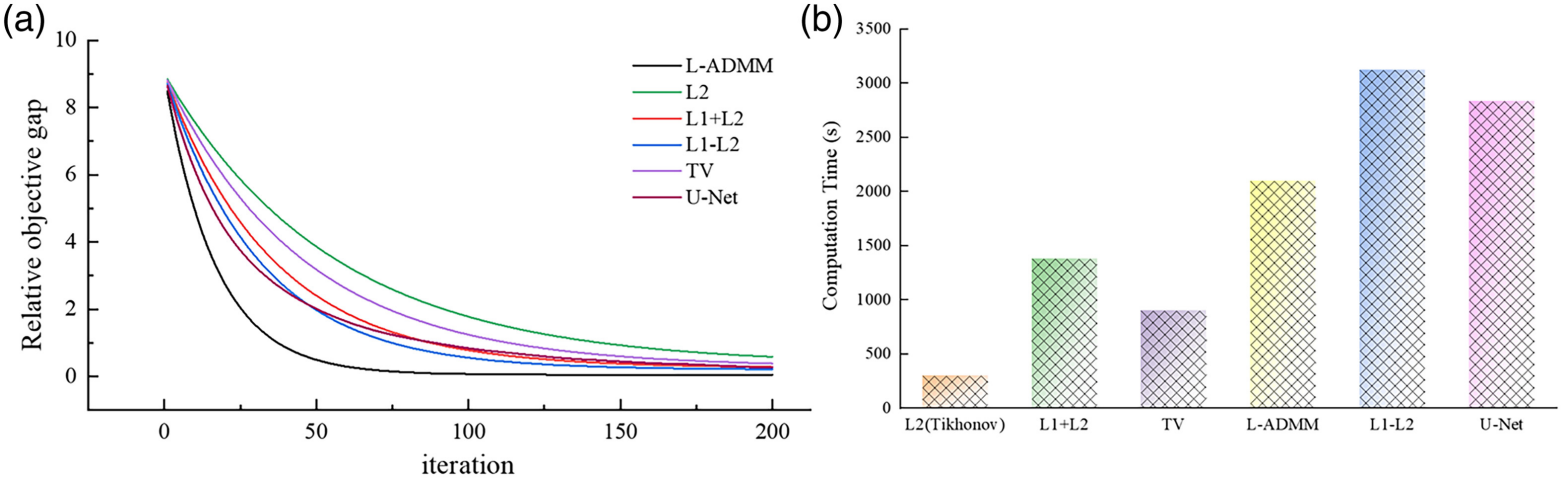
Comparison of convergence for various regularization methods and computation time of the vascular phantom under different reconstruction algorithms: (a) convergence comparison and (b) computation time.

At N=128, the L2 (Tikhonov) method [Fig. 8(n)] performs relatively poorly due to the ill-posed nature of sparse sampling, leading to multiple nonunique solutions and insufficient edge preservation. High-frequency details such as vessel boundaries are rapidly attenuated or even lost during optimization, and their response to increased sensor numbers is less effective than that of other algorithms. Although L1 + L2 and TV regularizations show some improvement, there remains a noticeable gap compared with L-ADMM in terms of detailed preservation and noise suppression, further verifying the superior performance of the proposed L-ADMM algorithm. In Fig. 9(a), the L-ADMM algorithm exhibits the fastest decline and reaches a stable state the earliest. It rapidly reduces the relative objective function value in the early iterations, achieving convergence earlier than other methods and showing superior convergence behavior. In Fig. 9(b) compared with the conventional L1–L2 method, L-ADMM significantly reduces computation time.

#### 4.4.3 Experimental results and analysis on the breast model

The real breast dataset was collected from the Medisch Spectrum Twente Hospital’s Breast Care Center at the Twente Medical Center, Netherlands, consisting of anonymized clinical PA images, as shown in Fig. 2(c). This figure presents a 2D PA reconstruction of a clinical breast model at a wavelength of 1064 nm. Based on the k-Wave toolbox, a numerical simulation was performed to generate forward data, which was then used for sparse reconstruction. The simulation assumed an acoustically homogeneous medium with a sound speed of 1540  m/s, using a circular transducer array composed of 12 arc-shaped arrays. The reconstruction region was located at the center, with a resolution of 0.4 mm and a signal length of 2054 sampling points. To evaluate the impact of sparse sampling, the number of transducers was set to N=32, 64, and 128, uniformly distributed along the array. Sparse reconstruction was performed based on the obtained forward signals, and the reconstructed images were compared with the preprocessed clinical reference images.

As shown in Table 3, L-ADMM achieved the best performance across all sampling densities. Under the low-density condition of N=32, L-ADMM reached a PSNR of 31.33 dB and an SSIM of 0.7972, representing significant improvements over L2 regularization by 9.66 dB and 17.3% and over TV regularization by 7.64 dB and 15.34%. The PSNR is 2.07 dB higher and the SSIM is 0.0315 higher than those of the U-Net. With N=64 sensors, the PSNR increased to 36.26 dB, corresponding to relative improvements of 32.29% and 23.04% over L2 and TV regularization, respectively. The SSIM reached 0.9665, improved by 0.1914 and 0.1082 compared with L2 and TV regularization, respectively, and increased by 13% and 8.28% compared with L1 + L2 and L1–L2 regularization. When the sensor number increased to N=128, PSNR and SSIM further increased to 46.70 dB and 0.9992, showing improvements of 13.69 dB and 10.9% compared with L1 + L2 regularization. These results indicate that the L-ADMM method exhibits remarkable noise robustness and edge-preserving capability under sparse sampling conditions, maintaining stable and high-quality reconstruction.

**Table 3:** Quantitative results of the breast model under different reconstruction algorithms.

Reconstruction algorithms	PSNR/dB				SSIM			
	N=32	N=64	N=128	N=384	N=32	N=64	N=128	N=384
TV	23.69	29.48	35.95	38.91	0.6912	0.8583	0.9463	0.9821
L2 (Tikhonov)	21.67	27.41	32.41	38.47	0.6791	0.7751	0.8578	0.9649
L1 + L2	24.76	29.96	33.01	37.25	0.7173	0.8626	0.9011	0.9754
L1–L2	26.19	34.10	38.67	39.10	0.7386	0.8926	0.9692	0.9600
U-Net	29.26	34.87	35.03	39.37	0.7657	0.8947	0.9781	0.9823
L-ADMM	**31.33**	**36.26**	**46.70**	**48.53**	**0.7972**	**0.9665**	**0.9992**	**0.9981**

Note: bold values indicate the best performance in each column.

**Fig. 10:**
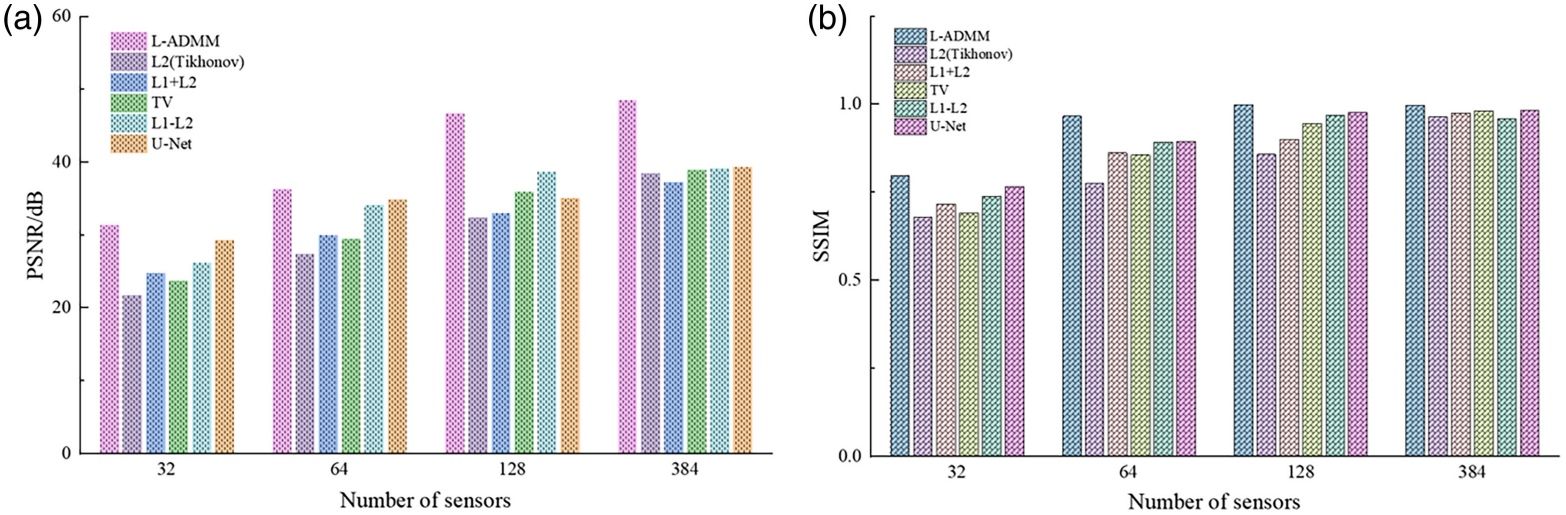
Quantitative evaluation of reconstruction performance using different algorithms under various sensor numbers: (a) comparison of PSNR under different reconstruction methods and (b) comparison of SSIM under different reconstruction methods.

For ease of comparison, Fig. 10 presents bar charts of PSNR and SSIM for different algorithms at various sampling densities. As shown, L-ADMM consistently achieves the highest metrics across all sensor counts. The proposed method significantly improves image quality under sparse-view conditions, demonstrating its robustness against artifacts and reliability in stable convergence.

**Fig. 11:**
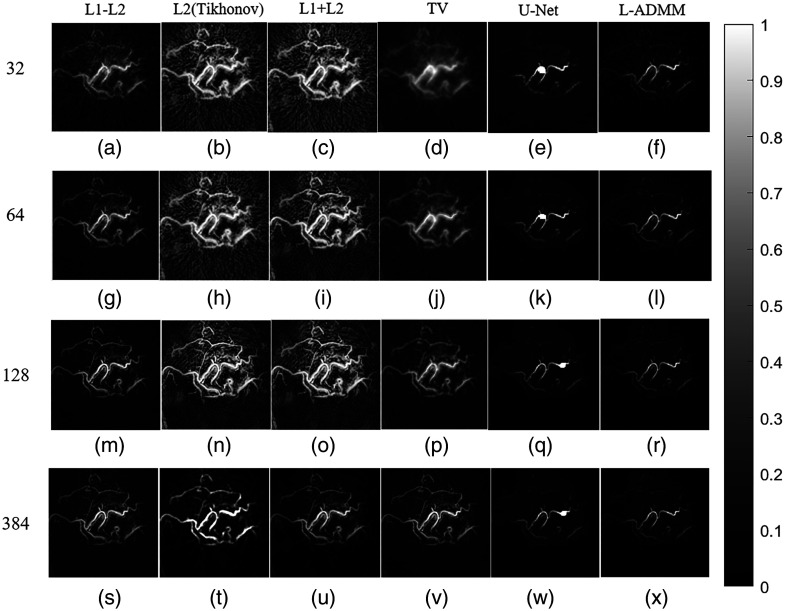
Reconstructed images of the breast model under different numbers of sensors. From top to bottom, the sensor counts are 32, 64, 128, and 384. From left to right, the reconstruction methods are L1–L2, L2 (Tikhonov), L1 + L2, TV, U-Net, and L-ADMM; gray scale bars are shown to indicate the dynamic range.

Figure 11 presents the reconstructed images of the breast model using different methods, providing a visual comparison of reconstruction performance. Under the low-density sampling condition of N=32, the L-ADMM reconstruction [Fig. 11(f)] preserves the contours of the main vascular branches, although the fine terminal vessels appear blurred. The background noise exhibits a granular distribution, and the pressure gradient of the photoacoustic signals is not fully restored.

**Fig. 12:**
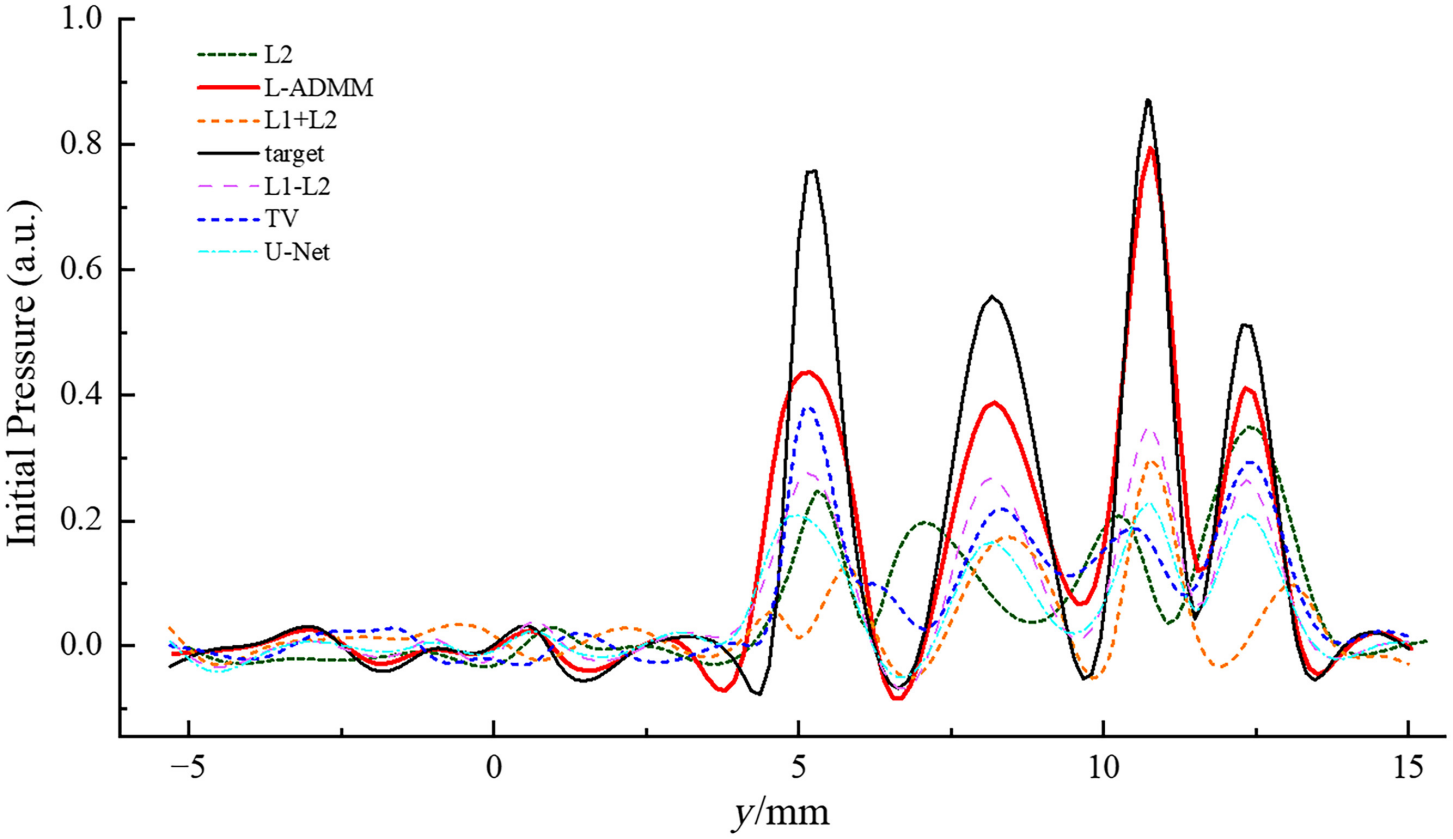
One-dimensional pressure distribution profiles of the reconstruction results under N=64 sensors.

In the L2 reconstruction [Fig. 11(b)], the main vascular structures show stripe-like artifacts, background noise is mixed with vascular signals, and vessel edges are blurred. The L1 + L2 method [Fig. 11(c)] improves branch continuity compared with L2, but scattered bright spots in the background noise are not fully suppressed, which masks fine vascular details. In the TV-regularized reconstruction [Fig. 11(d)], background noise appears as cloud-like interference, and the vascular structures are severely distorted, disrupting the continuity of the vessel signals. The L1–L2 regularization method [Fig. 11(a)] produces images that lack clarity in fine details; the branching structures and subtle features of the breast model are reconstructed with noticeable blurring, and the background contains considerable noise. The U-Net-based reconstruction [Fig. 11(e)] benefits from its deep feature extraction capability, allowing it to recover the overall vascular structure. However, its performance degrades in low-contrast and high-noise regions, where microvascular terminals and weak edges are poorly reconstructed. The network tends to over-smooth fine textures and occasionally introduces pseudo-structures, resulting in incomplete vessel connectivity and insufficient noise suppression in background regions. With N=64 sensors, the L-ADMM reconstruction [Fig. 11(l)] clearly resolves the main vascular branches and secondary branches. The algorithm effectively utilizes the limited data, balancing noise suppression with detail recovery. Fine terminal vessels (diameter ∼0.1  mm) become discernible, and the background noise changes from granular to uniformly low, resulting in a more complete reconstruction of the photoacoustic signal (Fig. 12).

In the L2 reconstruction [Fig. 11(h)], background noise is further suppressed, but some vascular details remain lost. The L1 + L2 reconstruction [Fig. 11(i)] achieves vascular detailed restoration comparable to L-ADMM, yet periodic noise persists in the background, causing subtle periodic intensity fluctuations. In the TV-regularized reconstruction [Fig. 11(j)], background noise is well suppressed, but the step-like artifacts at vessel edges are replaced by jagged artifacts; the overall vessel morphology is preserved, but local details remain blurred. The L1–L2 method [Fig. 11(g)] exhibits relatively more background noise interference. With N=128 sensors, the L-ADMM reconstruction [Fig. 11(r)] fully restores the main branches and multiple micro-branches, with fine terminal vessels clearly visible. The pressure gradient and phase information of the photoacoustic signals are completely preserved, and the reconstructed image outperforms the other five algorithms.

#### 4.4.4 Analysis of experimental results in the mouse ear vascular model

To further validate the effectiveness of the proposed method on animal experimental data, this study employs the publicly available photoacoustic vascular image dataset. The dataset was acquired using an OR-PAM system jointly developed by the Shenzhen Institutes of Advanced Technology and Qufu Normal University. It is specifically designed for photoacoustic microscopic imaging of vascular structures in mouse ears.

**Fig. 13:**
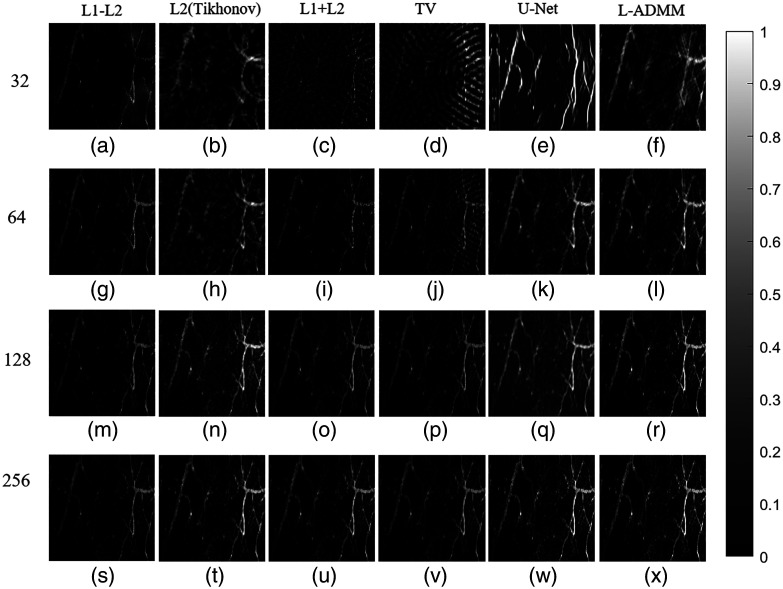
Reconstructed images of the mouse ear vascular model under different numbers of sensors. From top to bottom, the sensor counts are (N=32, 64, 128, and 256). From left to right, the reconstruction methods are L1–L2, L2 (Tikhonov), L1 + L2, TV, U-Net, and L-ADMM; gray scale bars are shown to indicate the dynamic range.

In the simulation, a one-dimensional linear-array photoacoustic transducer was used for signal reception. The array consists of 128 piezoelectric composite elements with a center-to-center spacing of ∼0.1  mm, resulting in a total aperture length of about 12.8 mm. During imaging, the dual-wavelength nanosecond pulsed laser—focused by a high-numerical-aperture objective—was scanned across the sample surface via a galvanometric mirror to excite localized photoacoustic signals. The linear-array transducer was incrementally translated along the vertical direction to achieve two-dimensional scanning. Figure 13 presents the reconstructed images of the mouse ear vascular model obtained by different reconstruction methods.

Under sparse sampling conditions (32 and 64 sensors), conventional analytical or convex optimization methods (such as L2, L1–L2, and TV regularizations) generally exhibit pronounced streak artifacts and structural blurring. Specifically, L2 regularization [Fig. 13(b)] leads to excessive smoothing, whereas L1–L2 [Fig. 13(a)] and TV [Fig. 13(d)] show slightly improved edge preservation but still suffer from strong noise-induced artifacts. By contrast, the U-Net model can better recover the main vascular structures; however, it tends to produce unstable over-enhancement or local discontinuities in fine details and intensity restoration. The proposed L-ADMM method [Fig. 13(f)] significantly suppresses streak artifacts and achieves superior boundary continuity compared with U-Net and TV. Under higher sampling conditions (128 and 256 channels), the reconstruction results of L-ADMM are almost identical to the ground truth, with clearly resolved microvascular structures and high signal contrast, demonstrating excellent robustness. Overall, the proposed L-ADMM algorithm maintains strong structural fidelity and noise suppression capability even under low-sampling conditions.

**Fig. 14:**
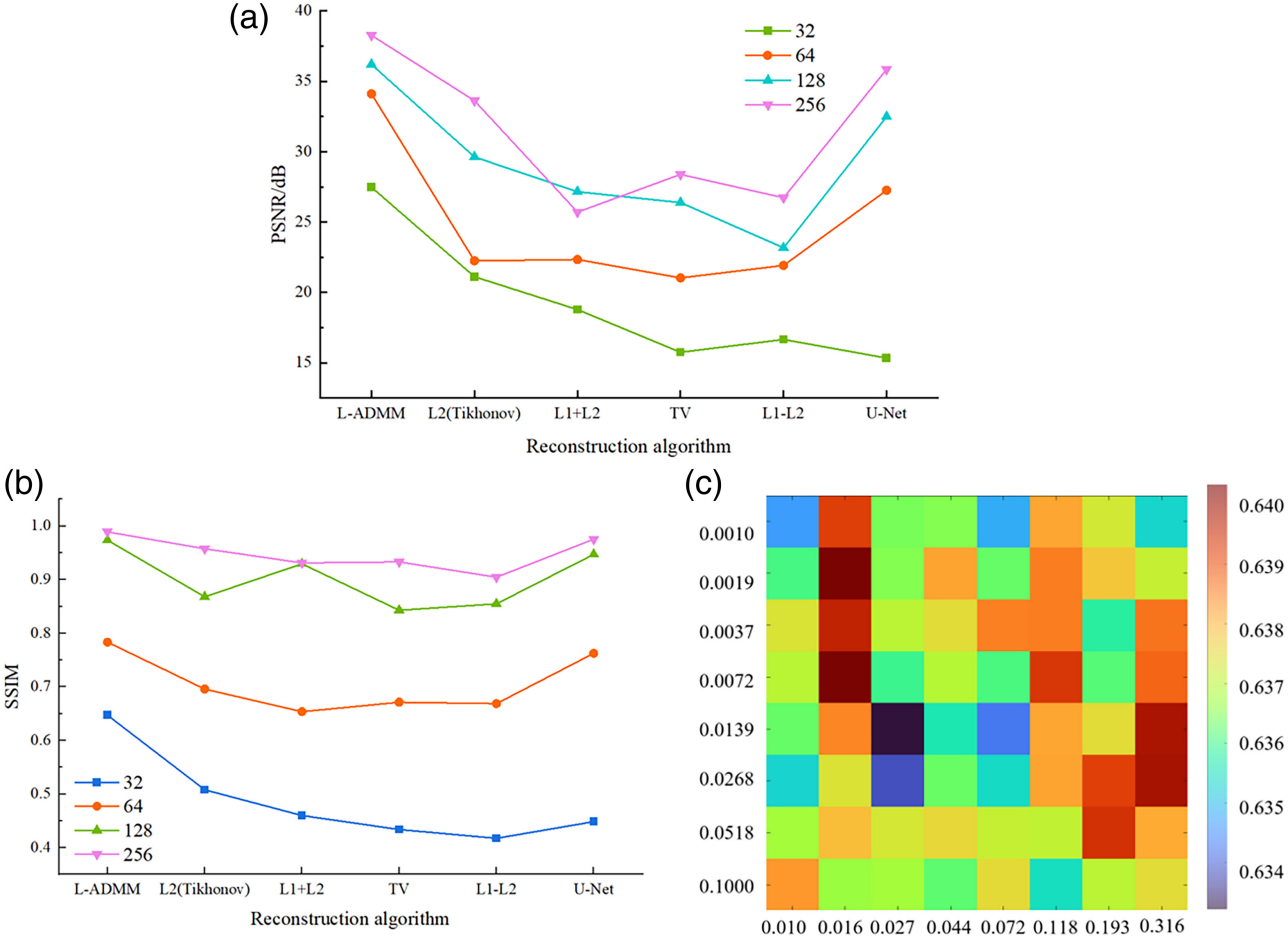
Quantitative comparison of reconstruction performance for the mouse ear vascular model under different algorithms: (a) PSNR comparison of various reconstruction methods at different numbers of sensors (N=32, 64, 128, and 256), (b) SSIM comparison of various reconstruction methods at different numbers of sensors, and (c) parameter sensitivity of L-ADMM reconstruction (SSIM) for the mouse ear vascular model at N=32, ρ=2, and SNR = 40 dB, showing the influence of $\lambda_{1}$ and $\lambda_{2}$ on reconstruction quality.

Figure 14(c) presents the SSIM sensitivity heat map of the L-ADMM algorithm for the mouse ear vascular model under the conditions of N=32 detector elements, iteration parameter ρ=2, and an SNR of 40 dB. The horizontal and vertical axes correspond to the regularization weights $\lambda_{1}$ (L1) and $\lambda_{2}$ (L2), respectively. The color intensity represents the SSIM value of the reconstructed image. As shown in the figure, the reconstruction performance of L-ADMM exhibits a certain degree of sensitivity to the regularization parameters; however, the overall variation range is relatively smooth, indicating good algorithmic stability. When $\lambda_{1}$ is relatively small (∼0.01 to 0.04) and $\lambda_{2}$ is within a moderate range (∼0.007 to 0.02), the reconstructed SSIM reaches relatively high values, as indicated by the light orange or yellow regions. This suggests that, within this parameter range, an appropriate balance between the L1 sparsity constraint and the L2 smoothness term is achieved, effectively suppressing noise while preserving the fine vascular structures.

When either $\lambda_{1}$ or $\lambda_{2}$ is excessively large (upper-right region), over-smoothing occurs, leading to a decrease in SSIM; conversely, when both are too small (lower-left region), the sparsity constraint becomes insufficient, resulting in increased residual noise and artifacts and a corresponding degradation in reconstruction quality. Overall, L-ADMM maintains stable reconstruction performance across a relatively wide parameter range. Furthermore, by appropriately selecting the ratio between $\lambda_{1}$ and $\lambda_{2}$, the structural fidelity and noise-suppression capability of the reconstructed images can be further optimized.

## 5 Conclusion

In this work, we proposed an L-ADMM-based photoacoustic tomography reconstruction algorithm, achieving efficient and stable reconstruction under sparse sampling conditions. Experimental results demonstrate that the method effectively preserves fine vascular and other sparse features while maintaining overall structural continuity and smoothness. It significantly alleviates common issues in conventional methods, such as edge blurring and artifacts. The proposed approach markedly improves image quality under sparse-view conditions and achieves reconstruction performance close to that of full-sampling, validating its effectiveness and superiority. Furthermore, this study extends the proposed reconstruction framework to linear-array transducer geometry, which is more practical for real-world photoacoustic imaging systems. The results demonstrate that the L-ADMM algorithm effectively mitigates the limited-view artifacts inherent to linear-array acquisition and achieves superior reconstruction quality with improved vessel continuity and noise suppression.

Compared with existing regularization methods, the proposed method shows clear advantages in objective metrics such as SSIM and PSNR. It enables high-quality imaging under low-cost hardware configurations and sparse sampling conditions, offering potential value for clinical applications such as early breast cancer screening and vascular disease diagnosis. Future research will focus on integrating physics-informed and data-efficient deep learning strategies into the proposed framework to further improve imaging speed and reconstruction accuracy while reducing dependence on large, specialized datasets.

## Data Availability

The breast model dataset used in this study was derived from the anonymous clinical data repository of Medisch Spectrum Twente Hospital, which provides publicly available photoacoustic breast imaging data for research and algorithm development. This dataset has been uploaded to the Figshare repository and is publicly accessible at https://figshare.com/s/030561cd4ca6f1c83256. In addition, the mouse ear vascular dataset was obtained from the publicly available Photoacoustic Vascular Image Dataset collected by the Shenzhen Institutes of Advanced Technology and Qufu Normal University using an Optical-Resolution Photoacoustic Microscopy system. The dataset is accessible at doi: 10.17632/dp5jgrkd6k.2. However, the code and data underlying the results presented in this paper are not publicly available at this time but may be obtained from the authors upon reasonable request.
